# Deciphering synergistic killing mechanisms of capsaicin combined with colistin against colistin-resistant *Acinetobacter baumannii* through metabolomics analysis

**DOI:** 10.1016/j.bbrep.2026.102740

**Published:** 2026-08-07

**Authors:** Mengjie Hou, Liying Yang, Changchao Huan, Tingting Guo

**Affiliations:** a Department of Microbiology, School of Basic Medicine, First Clinical Medical College, Faculty of Medicine, Yangzhou University, Yangzhou, 225001, China; b Jiangsu Key Laboratory of Zoonosis/Jiangsu Co-Innovation Center for Prevention and Control of Important Animal Infectious Diseases and Zoonoses, Yangzhou University, Yangzhou, 225001, China; c Jiangsu Key Laboratory of Integrated Traditional Chinese and Western Medicine for Prevention and Treatment of Senile Diseases, Yangzhou, 225001, China; d Institute of Agricultural Science and Technology Development, College of Veterinary Medicine, Yangzhou University, Yangzhou, 225001, China

**Keywords:** *Acinetobacter baumannii*, Untargeted metabolomics, Colistin, Capsaicin, Combination therapy

## Abstract

Colistin is increasingly used as last-line therapy for *A. baumannii* infections. This has led to the emergence of colistin-resistant strains of *A. baumannii*. Therefore, exploring novel approaches to fight colistin resistance in *A. baumannii* is urgently required. In our previous study, we reported that capsaicin combined with colistin could effectively circumvent colistin resistance in *A. baumannii*. Here, we employed untargeted metabolomics to explore the global metabolic perturbations of ATCC 19606-R induced by a combination of colistin (2 μg/mL) and capsaicin (32 μg/mL) compared to each of them acting alone at 1, 4, and 24 h.

Data revealed that the metabolic changes of ATCC 19606-R were initially driven by capsaicin (at 1 h) and then by colistin (at 4 h). The combination treatment induced remarkable metabolic perturbations at 4 h, which were mainly caused by colistin. This metabolic impairment continued for 24 h. Results also showed that vital metabolic pathways, such as those involved in carbohydrate, nucleotide, and amino acid metabolism, underwent significant changes. On the other hand, the combination treatment had a negligible effect on lipid metabolism. Data in this study suggest that a combination of capsaicin and colistin is a promising candidate for treating *A. baumannii* infections.

## Highlights

• Untargeted metabolomics reveals synergistic effect is initially triggered by CAP (1 h) and subsequently driven by COL (4 h).
• The combination disrupts nucleotide, amino acid, and carbohydrate metabolism, with negligible impact on lipid metabolism.
• The combination induces sustained metabolic impairment up to 24 h, superior to colistin or capsaicin monotherapy.
• These findings support capsaicin as a promising adjuvant to restore colistin efficacy against drug-resistant *A. baumannii.*

## 1 Introduction

*Acinetobacter baumannii* (*A. baumannii*) is one of the non-fermentative Gram-negative coccobacilli [1,2]. The bacterium is a nosocomial pathogen that can cause respiratory-related diseases [[3], [4], [5]] and meningitis [6,7], leading to relatively high mortality rates. Due to the extensive use of antibiotics, multidrug-resistant (MDR) strains of *A. baumannii* have widely emerged in medical settings [8,9]. Currently, bacterial multidrug resistance poses a significant cause for concern in medical facilities, with *A. baumannii* recognized as a difficult-to-control Gram-negative bacterium [10]. Therefore, the World Health Organization (WHO) has included *A. baumannii* in its “critical priority pathogens” list, for which new antibiotics are urgently needed [11].

Polymyxins (polymyxin A-E) are non-ribosomal cyclic oligopeptide antimicrobials. Of these, only polymyxins B and E (colistin) are applied clinically. However, colistin use was limited in the 1980s due to its nephrotoxicity [12] and acute toxicity [13]. Despite the toxicity concern, colistin re-emerged at the beginning of the 21st century as a treatment option for “superbugs” due to the limited availability of alternative antibiotic treatments [[14], [15], [16], [17], [18]]. Colistin exerts its antibacterial effect by targeting the bacterial cell membranes. The cationic colistin binds the anionic outer membrane lipopolysaccharides (LPS), changing the permeability and leading to leakage of the cellular contents and, finally, cell death [19]. Unfavorably, using colistin in clinical settings resulted in the emergence of colistin-resistant strains of *A. baumannii* [[20], [21], [22]]. The bacteria can resist colistin by modifying the LPS or lipid A phosphate groups of the cell membranes, thereby reducing binding to colistin [[23], [24], [25], [26]]. Colistin resistance and the failure of colistin monotherapy drive the search for synergistic colistin combinations that can kill colistin-resistant strains [27].

Compared with synthetic antibiotics, plant-derived compounds are less toxic and affordable [28] and, therefore, can be combined with antibiotics to enhance antimicrobial efficacy. Capsaicin (trans-8-methyl-N-vanillyl-6-nonenamide) is a homovanillic acid derivative identified as a secondary metabolite of chili peppers [29,30]. Besides making chili peppers hot, capsaicin also significantly influences the plant's immune system [29,31]. Studies have shown that capsaicin and its analogs exhibit several effects, such as relieving pain [[32], [33], [34], [35]], anti-inflammatory [36,37], inhibiting cell proliferation [[38], [39], [40], [41]], reducing fat accumulation, and being anti-obesity [37,42]. Capsaicin also reduced the virulence of methicillin-resistant *Staphylococcus aureus* (MRSA) by interfering with plasmid conjugation [43]. In addition, capsaicin could inhibit the cell invasion and hemolytic activity of *Streptococcus pyogenes* and achieve a bactericidal effect [31]. Furthermore, capsaicin suppressed the growth of *Porphyromonas gingivalis* by inhibiting biofilm formation, osteoclast precursor proliferation, and osteoclastogenesis [44]. Recently, we reported that capsaicin combined with colistin was effective against colistin-resistant *A. baumannii* [45]. The best antibacterial effect was achieved by combining 2 μg/mL of colistin with 32 μg/mL of capsaicin.

Metabolomics has emerged as an efficient systemic approach for identifying and quantifying vital intracellular metabolites in response to specified conditions at the network level [[46], [47], [48]]. Metabolic changes of colistin combined with other antibiotics have been reported in *A. baumannii* [47,[49], [50], [51]]. According to Maifiah and coworkers, colistin monotherapy could cause changes in fatty acid and phospholipid levels in as brief as 1 h, destroying the outer bacterial membrane [47]. In addition, Han and colleagues reported that colistin demonstrated a synergistic effect with sulbactam that significantly reduced nucleotide and amino acid contents in 4 h relative to the respective monotherapies [49].

Accordingly, the present investigation employed an untargeted metabolomics approach to analyze the mechanisms underlying the synergistic antimicrobial effect of the capsaicin and colistin combination against colistin-resistant *A. baumannii*.

## 2 Materials and methods

### 2.1 Bacteria and reagents

A reference strain of *A. baumannii* (ATCC 19606) was used in this study and was purchased from the American Type Culture Collection (ATCC), Manassas, USA. It was maintained at the Pathogenic Microbiology Laboratory, School of Basic Medicine, Yangzhou University. The reference strain showed colistin susceptibility with a minimum inhibitory concentration (MIC) of 0.5 μg/mL. A concentration of 2 μg/mL of colistin was used to induce resistance in the ATCC 19606-R strain. Colistin was purchased from Selleckchem (USA), and capsaicin was supplied by Tocris Bioscience (UK). Capsaicin stock solution (256 mg/mL) was prepared by dissolving 25.6 mg of capsaicin powder in 100 μL of absolute ethanol (100%). The solution was sterilized by membrane filtration (0.22-μM pore size) and stored at −20 °C until use. Colistin stock solution (128 mg/mL) was prepared by dissolving 100 mg of colistin powder in 781 μL of sterilized ddH_2_O, membrane filter sterilized using 0.22-μM filters, and stored at −20 °C.

### 2.2 Bacterial culture preparation for metabolomics assays

Before the assays, the ATCC 19606-R strain was inoculated onto nutrient agar plates and incubated at 37 °C for 16–18 h. A single colony of this culture was used to inoculate 10 mL of Cation-adjusted Mueller-Hinton Broth, CaMHB (Oxoid, Australia) and incubated for 18 h at 37 °C with shaking at 180 rpm. Afterward, the culture was diluted 1:100 with 200 mL of freshly prepared CaMHB in conical flasks. The diluted cultures were grown to an optical density at 600 nm (OD_600_) of 0.50 ± 0.02. The bacterial cultures were divided into four groups as follows: a drug-free group (served as a control), a group with 2 μg/mL of colistin, a group with 32 μg/mL of capsaicin, and a group with a combination of colistin (2 μg/mL) and capsaicin (32 μg/mL). Each group contained three biological replicates. The cultures were incubated at 37 °C for 1, 4, and 24 h. Samples were collected at each time point, and a total of 36 samples were subjected to metabolomic analysis.

### 2.3 Metabolite extraction for metabolomics studies

Following 1, 4, or 24 h of incubation, bacterial cultures were placed immediately in dry ice for quenching for 30 s to terminate metabolic processes. The cultures were normalized to an OD_600_ of 0.50 ± 0.02 with fresh CaMHB. Bacterial cells were collected from 3 mL of cultures by centrifugation at 4000 rpm at 4 °C for 10 min. Afterward, the samples stored at −80 °C were thawed on ice. After adding 500 μL of methanol aqueous solution (4: 1 ratio of methanol: Water, V/V) containing the endogenous reference, the cell samples were vortexed for 3 min. The samples were placed in liquid nitrogen for 5 min and in dry ice for another 5 min, followed by thawing on ice and 2-min vortexing for three freezing-thawing cycles to ensure efficient metabolite extraction. The samples were then centrifuged at 12000 rpm (at 4 °C), and 300 μL of supernatants were collected and kept for 30 min at −20 °C. Finally, a further 3-min centrifugation at 12000 rpm (at 4 °C) was performed, and 200 μL of supernatants were collected for the liquid chromatography-mass spectrometry (LC-MS) analysis.

### 2.4 LC-MS analysis of metabolites

The metabolite samples were analyzed using Triple TOF-6600 mass spectrometer (AB Sciex, USA) coupled to an LC20 ultra-high performance liquid chromatography, UPLC (Shimadzu, USA). The UPLC column was Waters ACQUITY UPLC HSS T3 C18 (1.8 μm, 2.1 × 100 mm). The column temperature was 40 °C. The injection volume was 2 μL, and the flow rate was 0.4 mL/min. The solvent system consisted of water (0.1% formic acid): acetonitrile (0.1% formic acid). The gradient procedure was set as follows: 95:5 V/V at 0 min, 10:90 V/V at 11 min, 10:90 V/V at 12 min, 95:5 V/V at 12.1 min, and 95:5 V/V at 14 min. Table 1 shows the conditions employed in positive/negative ion modes.

**Table 1:** Gradient conditions of the mobile phase for the chromatographic column.

time(min)	flow rate(mL/min)	A(%)	B(%)
0.0	0.4	95	5
11.0	0.4	10	90
12.0	0.4	10	90
12.1	0.4	95	5
14.0	0.4	95	5

### 2.5 Chromatography and mass spectrometry data acquisition conditions

Chromatographic separation was performed using a Waters ACQUITY UPLC HSS T3 C18 column (1.8 μm, 2.1 mm × 100 mm). The mobile phase consisted of solvent A (ultrapure water containing 0.1% formic acid) and solvent B (acetonitrile containing 0.1% formic acid). The column temperature was maintained at 40 °C with a constant flow rate of 0.40 mL/min. The injection volume was set to 2 μL for each sample. The gradient conditions of the mobile phase for the chromatographic column were shown in Table 1 and the positive and negative ion mode mass spectrometry conditions were shown in Table 2.

**Table 2:** Positive and negative ion mode mass spectrometry conditions.

conditions	ESI+	ESI-
IonSpray Voltage	5500	−4500
Ion Source Gas1	50	50
Declustering Potential(DP)	60	−60
Curtain Gas	35	35
Temperature	550	550
Ion Source Gas2	60	60
Collision Energy	30	−30

### 2.6 Data processing, bioinformatics, and statistical analyses

The raw LC-MS data were converted to mzML format using the ProteoWizard software. The XCMS program was then utilized to extract and align peaks and correct retention times separately. In addition, the peak area was corrected by the “SVR” approach. Samples with a peak detection rate of < 50% were eliminated. Afterward, the self-built database of the laboratory, metDNA, AI database, and integrated public databases were searched to acquire metabolic identification profiles. In this study, univariate and multivariate statistical analyses were integrated to screen differential metabolites from multiple dimensions according to the data characteristics, so as to achieve accurate identification of differential metabolites.Univariate statistical analysis was performed using parametric and non-parametric tests, while multivariate statistical analysis principally included principal component analysis (PCA) and orthogonal partial least squares discriminant analysis (OPLS-DA). Variable importance in projection (VIP) values derived from the established OPLS-DA model were initially adopted to screen metabolites with significant intergroup differences among different varieties or tissues. Furthermore, P-values obtained from univariate analysis (Student's t-test for two-group comparisons and one-way ANOVA for multi-group comparisons) and fold change (FC) values were combined for the further validation and screening of differential metabolites.Differential metabolites were comprehensively screened by integrating FC values, P-values, and OPLS-DA VIP values to ensure the reliability and accuracy of screening results.Metabolites with FC ≥ 2.0 or FC ≤ 0.5 were defined as significantly differential.

## 3 Results

### 3.1 Metabolomics analysis of *A. baumannii* ATCC 19606-R treated with colistin and/or capsaicin

**Fig. 1:**
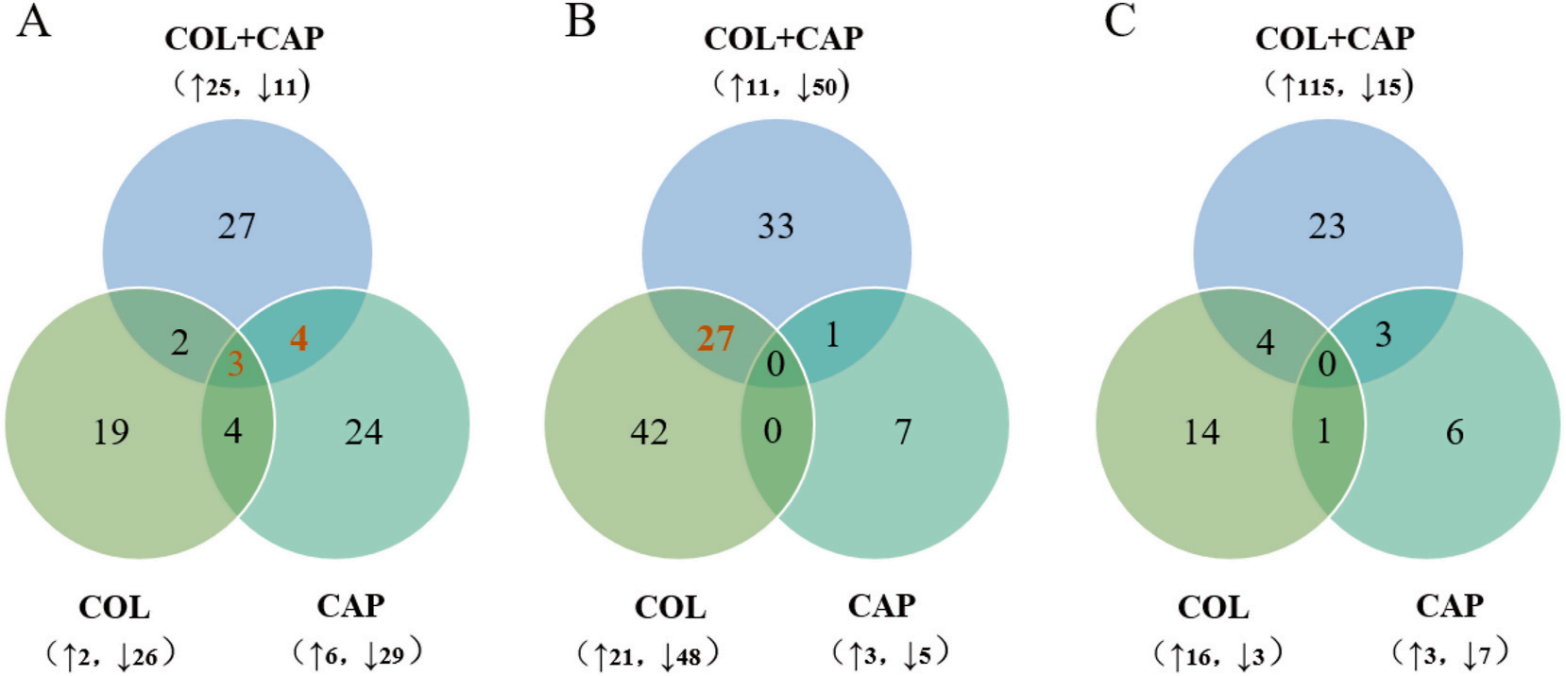
Multivariate and univariate regression on global changes in metabolites. Venn diagrams showing significant differential metabolites number below every treatment at (A) 1 h, (B) 4 h, and (C) 24 h. COL, colistin; CAP, capsaicin; COL + CAP, colistin and capsaicin combined (FDR ≤ 0.05 by one-way ANOVA; *p* ≤ 0.05 upon Fisher's LSD).

Based on our previous published work and OD_600_ of *A. baumannii* treated with different drug regimens at 1h,4h,and 24h(Supplementary Fig. S1), the combination of capsaicin and colistin achieved markedly enhanced bactericidal efficacy against *A.baumannii* at 24 h.These results validated the rationality of drug concentrations and sampling timepoints adopted in untargeted metabolomic. Metabolomics analyses revealed 1968 presumptively identified metabolites (Table 3), including 382 from amino acid metabolism, 40 carbohydrates, 8 coenzyme factors and vitamins, 222 lipids, and 99 involved in nucleotide metabolism. Typically, 471 of these metabolites, accounting for 23.9% coverage of all metabolites detected in the ATCC 19606-R strain, were associated with the KEGG pathway. For the combined quality control samples, the median relative standard deviation (RSD), which showed a minimal technical difference, was 1.135%, well within the acceptable limits for metabolomics studies. As illustrated by Venn diagrams (Fig. 1), the perturbation resulting from the colistin and capsaicin combination occurred in 36 (11 down-regulated and 25 up-regulated), 61 (50 down-regulated and 11 up-regulated), and 30 (15 down-regulated and 15 up-regulated) differentially expressed metabolites at 1, 4, and 24 h, respectively. Treatment with colistin alone led to perturbation in 28 (26 down-regulated and 2 up-regulated) and 69 (48 down-regulated and 21 up-regulated) differential metabolites at 1 and 4 h, respectively. Significantly decreased levels of perturbation were recorded at 24 h, with just 19 metabolites showing significant differential expression (3 down-regulated and 16 up-regulated). Capsaicin treatment demonstrated higher perturbations (29 down-regulated and 6 up-regulated) at 1 h but less effect at 4 and 24 h, with only eight and ten significant metabolite changes, respectively, compared to colistin alone or the colistin combined with capsaicin.

**Table 3:** Statistical summary of identified metabolites in metabolomic analysis.

-	All	Positive	Negative
Number of metabolites	3659	2079	1580
Number of secondary metabolite identifications	**1968**	1165	803

Data also revealed that the different treatments shared only three metabolic perturbation intermediates in common at 1 h, while no common perturbations were observed at 4 or 24 h. In addition, the colistin and capsaicin combination showed 44% of common metabolic variations with colistin treatment at 4 h while less at 1 and 24 h. Likewise, combined treatment shared about 20% of metabolic variations with capsaicin alone at 1 h while less at 4 and 24 h. These results suggest that the synergy achieved by colistin-capsaicin combined treatment was primarily triggered by capsaicin at 1 h and driven by colistin at 4 h (Fig. 1).

### 3.2 KEGG analysis

**Fig. 2:**
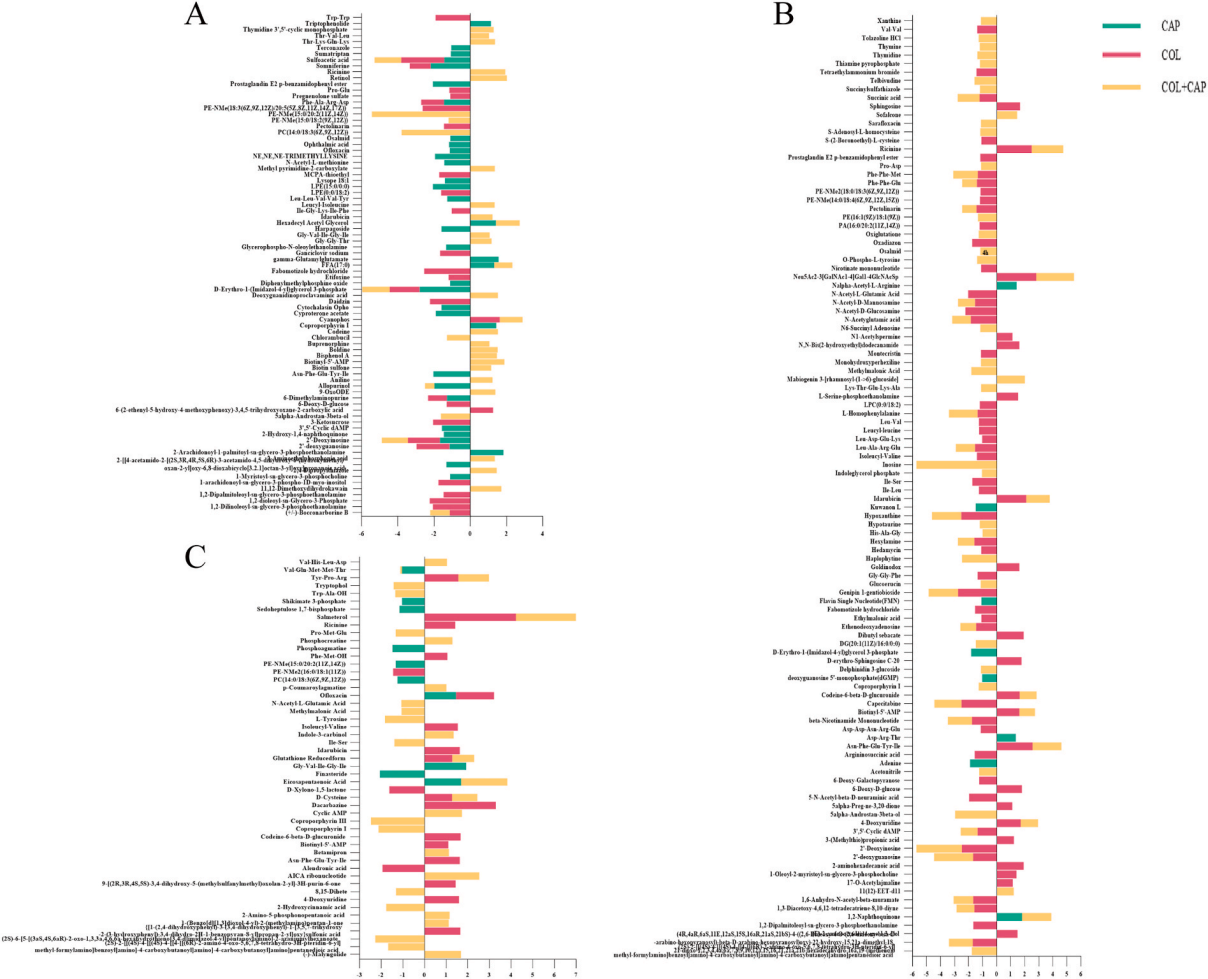
KEGG enrichment analysis. Perturbed pathways identified by enrichment analysis in *A. baumannii* ATCC 19606-R due to single colistin (COL) or capsaicin (CAP) treatment or in combination (COL + CAP) at (A) 1 h, (B) 4 h, and (C) 24 h (FDR < 0.05 and *p* < 0.05).

**Fig. 3:**
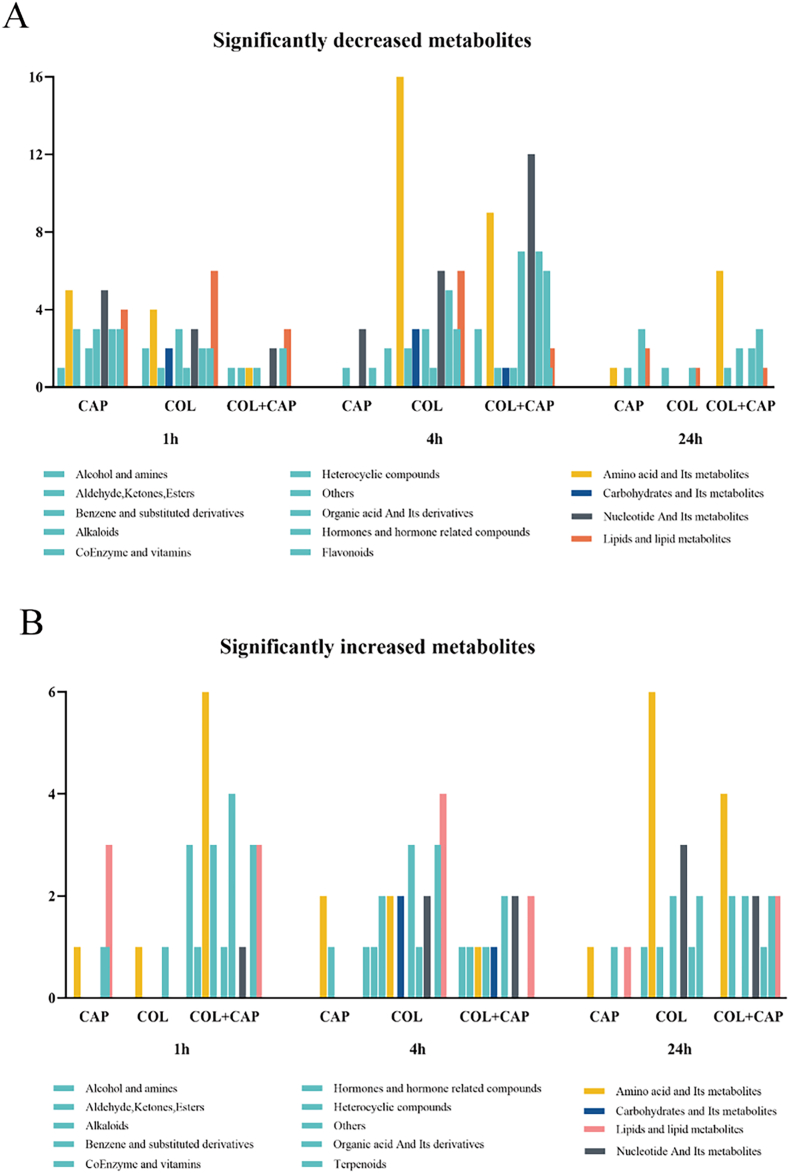
Numbers of markedly up-regulated (A) and down-regulated (B) metabolites related to main metabolic pathways in *A. baumannii* ATCC 19606-R due to colistin (COL) and capsaicin (CAP) treatments alone or in combination (COL + CAP) at 1, 4, and 24 h (FDR < 0.05, *p* < 0.05).

The metabolites were further examined and analyzed according to the Kyoto Encyclopedia of Genes and Genomes (KEGG). Data revealed that the metabolites were enriched, suggesting that several critical biochemical pathways in *A. baumann*ii ATCC 19606-R were significantly affected within 24 h of treatment with colistin or capsaicin alone or in combination (Fig. 2). These biochemical pathways were associated with amino acid, nucleotide, carbohydrate, and lipid metabolism. The metabolic changes were more significant at 4 h and continued until 24 h. At 4 h post-treatment, more metabolite changes were recorded for colistin single treatment and colistin and capsaicin combination groups, suggesting that the metabolic perturbation at 4 h was mainly driven by colistin (Fig. 2, Fig. 3). At 24 h, only a few metabolite changes caused by colistin or capsaicin alone were observed, while most of the changes were in the combined treatment group, proving that a combination of colistin and capsaicin was more effective than each of the two agents acting alone against *A. baumannii* ATCC 19606-R.

### 3.3 Analyses of dysregulated lipids and lipid metabolites

**Fig. 4:**
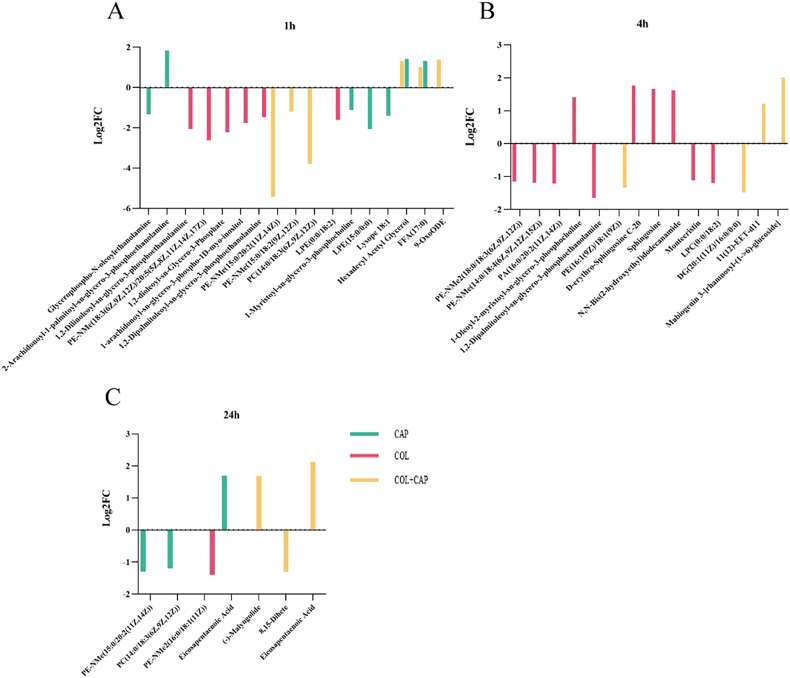
Perturbations in lipid metabolites. The lipid metabolism of *A. baumannii* ATCC 19606-R was significantly altered by colistin or capsaicin alone or in combination after 1 h, 4 h, and 24 h of treatment(FDR < 0.05, *p* < 0.05).

Metabolomics analysis of lipids revealed that at 1 h, colistin and capsaicin combination treatment caused a decrease in the abundance of glycerophospholipids, such as PE-NMe (15:0/20:2(11Z,14Z)), which belongs to phosphatidylethanolamine, PE (log2 FC = −5.4), PE-NMe (15:0/18:2(9Z,12Z)), which belongs to phosphatidylethanolamine, PE (log2 FC = −1.2), and PC (14:0/18:3(6Z,9Z,12Z)), which belongs to glycerophosphocholines, PC (log2 FC = −3.8) (Fig. 4). Treatment with colistin alone was not significantly different from the combination treatment at 1 h. At 4 h, the combination treatment had a minimal influence on lipid metabolism, causing decreased abundance only in PE (16:1(9Z)/18:1(9Z)), log2 FC = −1.3. In contrast, treatment with colistin combined with capsaicin did not affect lipid metabolism at 24 h, while capsaicin alone caused a decline in the abundance of PE-NMe (15:0/20:2(11Z,14Z)) and PC (14:0/18:3(6Z,9Z,12Z))(≥−1.0-log2-fold; *p* ≤ 0.05) and colistin decreased the abundance of PE-NMe2 (16:0/18:1(11Z)) (≥−1.0-log2-fold; *p* ≤ 0.05) at the same time point. The previous studies suggest that colistin targets the LPS on the outer membrane of Gram-negative bacteria, causing lipid metabolic impairment and outer membrane rupture, allowing increased colistin uptake and, eventually, cell death [52]. Notably, a few variations in lipid metabolites were observed in the present study. The fact that *A. baumannii* ATCC 19606-R used in this study was colistin-resistant and could modify the LPS or lipid A phosphate groups in cell membranes, causing reduced colistin binding, may explain this observation.

### 3.4 Analysis of perturbations in amino acid metabolism

**Fig. 5:**
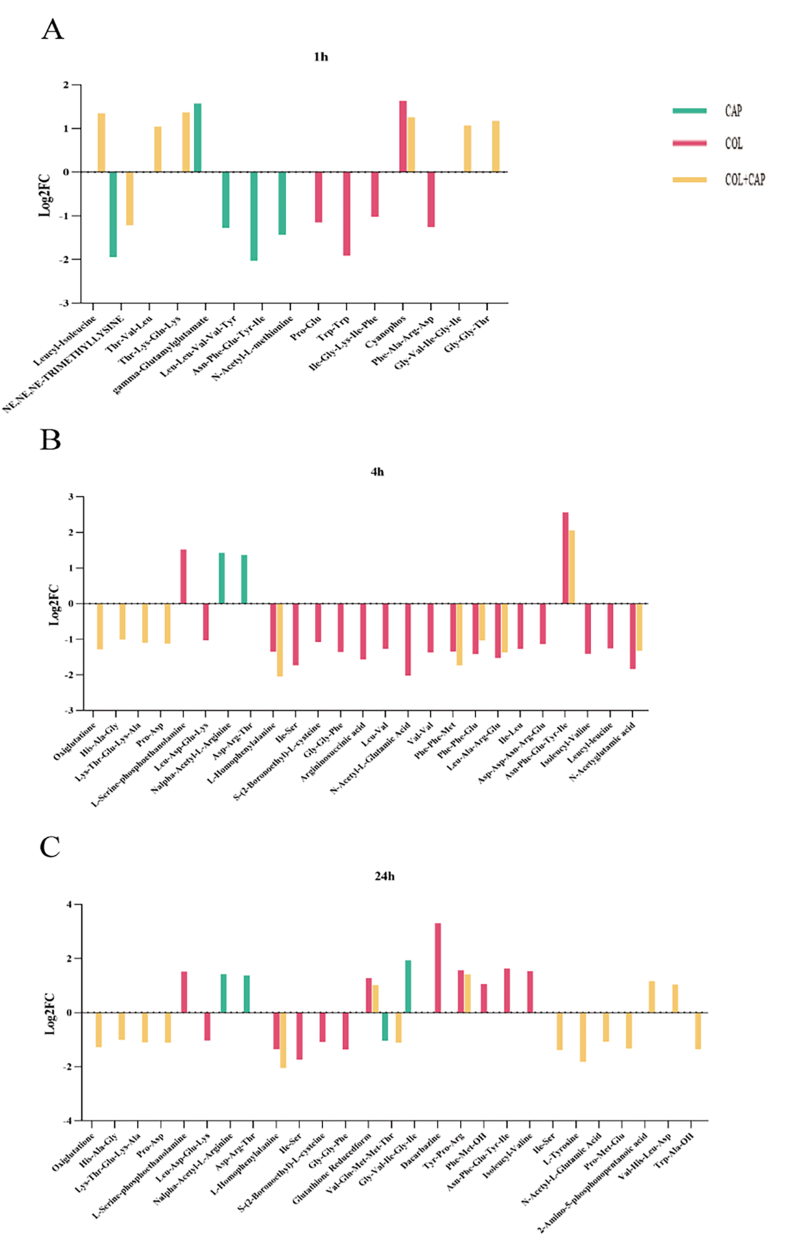
The significantly affected amino acid metabolites produced by *A. baumannii* ATCC 19606-R after 1 h, 4 h, and 24 h of treatment with colistin or capsaicin alone or in combination(FDR < 0.05, *p* < 0.05).

Treatment with colistin alone significantly suppressed the metabolism of only four peptides at 1 h and 17 peptides at 4 h, while it did not affect amino acid metabolism at 24 h. On the other hand, capsaicin treatment had no significant effect on amino acid metabolism at 4 and 24 h, inducing only two amino acid alterations at both time points (Fig. 5). Noteworthy, metabolites involved in the anabolic pathway of arginine include N-acetyglutamic acid, argininosuccinic acid, N-acetyl-l-Glutamic Acid, p-coumaroylagmatine, and phosphocreatine. The effects of different treatments on these amino acid metabolites were most significant at 4 h and 24 h of treatment. At 4 h, colistin alone caused significant down-regulation in N-acetyglutamic acid, argininosuccinic acid, and N-acetyl-l-glutamic Acid, while the combination treatment induced N-acetyglutamic acid down-regulation (Fig. 5).

### 3.5 Analysis of nucleotide metabolic changes

**Fig. 6:**
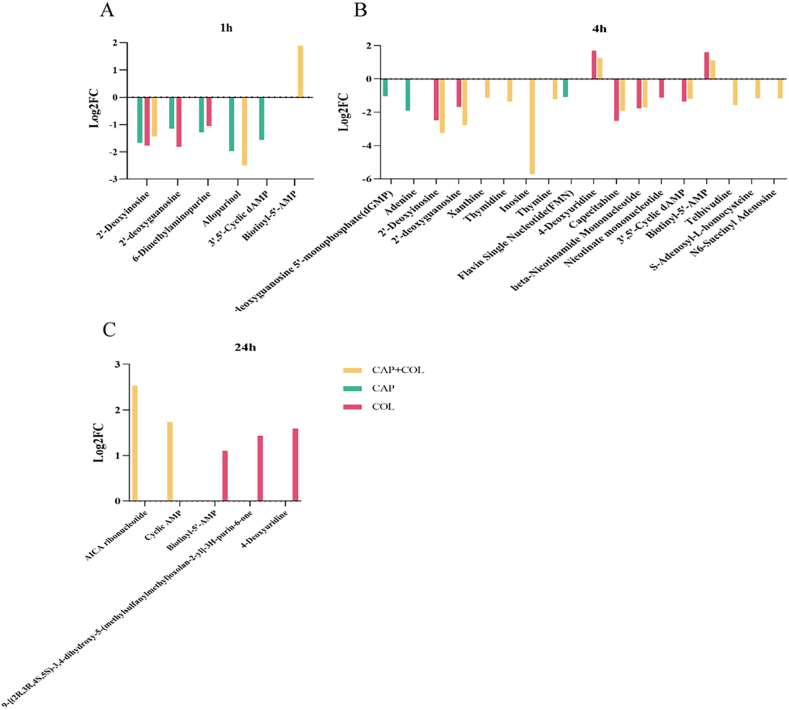
The significantly affected nucleotide metabolites developed by *A. baumannii* ATCC 19606-R after 1 h, 4 h, and 24 h of treatment with colistin or capsaicin alone or in combination(FDR < 0.05, *p* < 0.05).

The colistin and capsaicin combination markedly depleted nucleotides in *A. baumann*ii ATCC 19606-R, especially at 4 h. The depletion level decreased at 1 h and disappeared at 24 h. Eleven nucleotides were markedly down-regulated by the combination treatment at 4 h, including thymine, xanthine, thymidine, dAMP (≥−1.0-log2-fold; *p* ≤ 0.05), methylmalonic acid, thymidine, inosine, thymine xanthine, 2′-deoxyinosine, 2′-deoxyguanosine, and hypoxanthine (Fig. 6). Capsaicin alone exhibited a lower effect, down-regulating three fundamental nucleotides (adenine, dGMP, and flavin single nucleotide) at the same time point. A single treatment with either capsaicin or colistin could down-regulate 2′-deoxyinosine and 2′-deoxyguanosine at 1 h.

## 4 Discussion

Novel colistin combination therapy against *A. baumannii* is required to extend the therapeutic impact of colistin while minimizing the toxic effect. We previously reported that capsaicin combined with colistin synergistically killed *A. baumannii* [45]. Here, we utilized LC-MS/MS-based metabolomics to explore the exact mechanism of action by investigating the metabolic changes in colistin-resistant *A. baumannii* strains upon exposure to a colistin combination with capsaicin. As far as we know, this is the first study to explore the synergy mechanism of capsaicin and colistin combinations in killing *A. baumannii* based on an untargeted metabolomics approach.

Colistin exerts its antibacterial activity by destroying the bacterial outer membranes [53]. Phosphatidylethanolamine (PE), cardiolipin, and phosphatidylglycerol (PG) represent the main lipid constituents of the cell envelope of Gram-negative bacteria. These lipids play vital roles in keeping the bacterial cell intact and ensuring survival under stress [54]. With this in mind, we first analyzed the effects of colistin and capsaicin alone or in combination on lipids and lipid metabolites. Surprisingly, only three glycerophospholipids showed a marked decline in abundance upon exposure of *A. baumannii* to the combination treatment at 1 h. No effect was observed at 4 or 24 h of treatment. This negligible perturbation in lipid metabolism can be explained by the fact that *A. baumannii* ATCC 19606-R was colistin-resistant and could alter its LPS or lipid A phosphate groups in the membranes, resulting in a reduced binding affinity to colistin.

As an active component of chili peppers, capsaicin demonstrated several beneficial properties, such as antioxidant, anticancer, and antimicrobial activities. The antimicrobial activity of capsaicin has drawn wide attention in recent years, but the exact mechanism of capsaicin synergy with colistin remains unknown [55]. Previous studies suggested that capsaicin inhibited bacterial growth by reducing membrane stability due to its hydrophobic nature [56]. Other studies claimed that capsaicin could destroy bacterial cell membranes while inhibiting cell development-related genes [57]. In the present study, capsaicin could inhibit bacterial cell growth by inhibiting RNA/DNA metabolism and inducing perturbations in the amino acid metabolites.

Notably, the global metabolic perturbation triggered by combined treatment at 1 h represents an indispensable early sensitization event underlying capsaicin-colistin synergy, which cannot be reproduced by capsaicin or colistin monotherapy alone. At this early time point, the combinatorial regimen simultaneously disturbs three functionally critical pathways: glycerophospholipid metabolism, aromatic amino acid-redox homeostasis, and nucleotide precursor biosynthesis. Mild depletion of outer membrane glycerophospholipids partially dismantles the modified lipid A barrier of colistin-resistant *A. baumannii*, diminishing electrostatic repulsion against cationic colistin and promoting drug penetration [58]. Meanwhile, disordered arginine metabolism disrupts cellular antioxidant systems and elevates intracellular oxidative stress, further sensitizing bacteria to colistin-mediated membrane damage [59]. This dual metabolic priming at 1 h establishes a susceptible physiological state, which enables colistin to induce profound, irreversible collapse of nucleotide and central amino acid metabolism at 4 h and 24 h. Collectively, the coordinated multi-pathway metabolic remodeling initiated at 1 h serves as the upstream metabolic basis for the subsequent robust synergistic bactericidal activity observed in our study.

In conclusion, untargeted metabolomics revealed that the capsaicin-colistin combination exerts synergistic bactericidal activity via sequentially disrupting core bacterial metabolic networks. Capsaicin first drives early metabolic perturbation at 1 h, while colistin dominates profound, sustained disruption of amino acid and nucleotide metabolism at 4 h and 24 h; these coordinated metabolic impairments jointly underpin the combinatorial antibacterial synergy.

## Institutional review board statement

Not applicable.

## Funding

This work was supported by the Postgraduate Research & Practice Innovation Program of Jiangsu Province (Project No.: KYCX25_4097).

## CRediT authorship contribution statement

**Mengjie Hou:** Data curation, Funding acquisition, Methodology, Writing – original draft. **Liying Yang:** Data curation, Formal analysis, Project administration, Resources. **Changchao Huan:** Supervision. **Tingting Guo:** Writing – review & editing.

## Declaration of competing interest

This work was supported by the Jiangsu Provincial Postgraduate Research and Practical Innovation Program (Grant No. KYCX25_4097). The funders had no role in the design of the study; in the collection, analyses, or interpretation of data; in the writing of the manuscript; or in the decision to publish the results.

The authors declare that there are no competing interests.

## Data Availability

Data will be made available on request.
