# Supplementary material for: Deciphering synergistic killing mechanisms of capsaicin combined with colistin against colistin-resistant Acinetobacter baumannii through metabolomics analysis
Source: Biochem Biophys Rep. 2026 Aug 7;47:102740. doi: 10.1016/j.bbrep.2026.102740 (PMC13476486; doi:10.1016/j.bbrep.2026.102740)
Supplement: Supplementary file 1 [file mmc1.docx]

Figure S1.Optical density (OD₆₀₀) of *A. baumannii* treated with different drug regimens at 1h,4h,and 24h. Data were shown as mean ± SD of three replicates, and nonparametric one-way ANOVA was used to assess the statistical significance (**p* < 0.05, ***p* < 0.01), and ‘‘ns’’ indicates no significant difference.
